# Quercetin Alleviates Lipopolysaccharide-Induced Cell Damage and Inflammation via Regulation of the TLR4/NF-κB Pathway in Bovine Intestinal Epithelial Cells

**DOI:** 10.3390/cimb44110356

**Published:** 2022-10-27

**Authors:** Xiaoxiao Gong, Yinghao Huang, Qianbo Ma, Maocheng Jiang, Kang Zhan, Guoqi Zhao

**Affiliations:** 1 Institute of Animal Culture Collection and Application, College of Animal Science and Technology, Yangzhou University, Yangzhou 225009, China; 2 Institutes of Agricultural Science and Technology Development, Yangzhou University, Yangzhou 225009, China; 3 Joint International Research Laboratory of Agriculture and Agri-Product Safety, The Ministry of Education of China, Yangzhou University, Yangzhou 225009, China

**Keywords:** quercetin, fruit and vegetable extracts, antioxidant, barrier function, anti-inflammatory

## Abstract

Acute diarrhoea and intestinal inflammation represent one of the most prevalent clinical disorders of milk production, resulting in enormous annual financial damage for the dairy sector. In the context of an unsatisfactory therapeutic effect of antibiotics, the natural products of plants have been the focus of research. Quercetin is an important flavonoid found in a variety of plants, including fruits and vegetables, and has strong anti-inflammatory effects, so it has received extensive attention as a potential anti-inflammatory antioxidant. However, the underlying basis of quercetin on inflammatory reactions and oxidative tension generated by lipopolysaccharide (LPS) in bovine intestinal epithelial cells (BIECs) is currently unexplained. This research aimed to determine the influence of quercetin on LPS-induced inflammatory reactions, oxidative tension, and the barrier role of BIECs. Our findings demonstrated that BIEC viability was significantly improved in LPS-treated BIEC with 80 μg/mL quercetin compared with the control group. Indicators of oxidative overload and genes involved in barrier role revealed that 80 μg/mL quercetin efficiently rescued BIECs from oxidative and barrier impairment triggered by 5 μg/mL LPS. In addition, the mRNA expression of pro-inflammatory cytokines TNF-α, IL-1β, and IL-6, as well as chemokines CXCL2, CXCL5, CCL5, and CXCL8, was diminished in LPS-treated BIECs with 80 μg/mL quercetin compared with LPS alone. Furthermore, the mRNA expression of toll-like receptor 4 (TLR4), CD14, myeloid differential protein-2 (MD2), and myeloid differentiation primary response protein (MyD88) genes associated with the TLR4 signal mechanism was markedly reduced by the addition of quercetin to LPS-modulated BIECs, indicating that quercetin can suppress the TLR4 signal mechanism. We performed Western blotting on the NF-κB signalling mechanism and compared it with immunofluorescence to further corroborate this conclusion. The LPS treatment enhanced the proportions of p-IκBα/GAPDH and p-p65/GAPDH. Compared with the LPS-treated group, quercetin administration decreased the proportions of p-IκBα/GAPDH and p-p65/GAPDH. In addition, immunofluorescence demonstrated that quercetin greatly reduced the LPS-induced nuclear translocation of NF-κB p65 in BIECs. The benefits of quercetin on inflammatory reactions in LPS-induced BIECs may be a result of its capacity to inhibit the TLR4-mediated NF-κB signalling mechanism. These findings suggest that quercetin can be used as an anti-inflammatory reagent to treat intestinal inflammation induced by LPS release.

## 1. Introduction

Enterotoxigenic Escherichia coli (ETEC) is a major pathogen that can invade and cause disease in various species, including humans and animals [1]. As a dangerous pathogenic bacteria, ETEC limits the quality of dairy products and yield, which can cause significant economic losses to cattle farmers [2,3]. The colonization of the intestinal tract by ETEC secretes enterotoxins [4]. The accumulation of enterotoxins in the intestine causes damage to intestinal epithelial cells and, subsequently, induces diarrhoea and an inflammatory response [5]. LPS is an endotoxin ingredient produced by Gram-negative bacteria that stimulates a robust immune reaction in the intestine [4]. Intestinal epithelial cells are major components of the intestine’s mucosa and are targets during inflammation of the intestine [6]. Epithelia are the first line of defence against pathogenic microorganisms [7]. In the end, the proliferation of pathogenic microbes weakens the mechanical barrier action of intestinal epithelial cells, allowing various harmful elements, such as LPS, to invade the intestines [8]. TLR4-mediated stimulation of the NF-κB signal mechanism induces the production of pro-inflammatory cytokines and chemokines in response to the LPS-LBP-CD14-MD2 compound, according to an earlier study [9]. These inflammatory modulators then participate in cellular homeostasis and exert general effects [10]. Thus, in this study, LPS was employed to construct a cell inflammation model to study the protective mechanism of quercetin on inflammatory responses in LPS-induced BIEC.

In the past few years, antibiotics have become one of the most important approaches to managing inflammation [11]. Unfortunately, this therapy frequently results in antibiotic persistence and bacterial drug resistance [12]. Thus, the production of other medications is necessary for the management of intestinal inflammation. In previous reports, the flavonoids in plant extracts have been shown to have great medical value for inflammation [13]. In a previous study [14], it was found that the flavone quercetin is one of the most abundant polyphenolic compounds present in many plant sources and has been ascribed antioxidant, anti-inflammatory, and anti-apoptotic features [15,16]. In addition, quercetin can be converted into other conjugated metabolites of quercetin under the action of microorganisms [17]. These metabolites also show various pharmaceutical activities, including antioxidant and anti-inflammatory [18,19]. Published studies have similarly demonstrated quercetin to be remarkably nontoxic in animals and humans and to exert a protective effect on the animal gastrointestinal tract [20]. In prior laboratory studies [15], we discovered that quercetin lowers the production of IL-1β, IL-6, and TNF-α in LPS-treated human oral fibroblasts. The research on quercetin versus H_2_O_2_-induced inflammation revealed the anti-inflammatory properties of quercetin in endothelial cells of the human umbilical vein [21]. However, it is not clear whether quercetin can protect BIEC from inflammatory damage.

We anticipated that quercetin could modulate the inflammatory response of LPS-stimulated BIECs by limiting the TLR signalling mechanism. This research examines the ameliorating effect of quercetin on LPS caused by intestinal epithelial cell inflammation in dairy cows, as well as its potential methods, in order to establish a theoretical foundation for subsequent studies and the progression of quercetin-based new synthetic medications to treat intestinal inflammation.

## 2. Materials and Methods

### 2.1. Bovine Intestinal Epithelial Cell Culture

BIECs were obtained from the Institute of Animal Culture Collection and Development at Yangzhou University in China. The establishment was based on previous studies [22]. BIECs were cultured in DMEM/F12 media (Gibco, Grand Island, NY, USA) along with 10% foetal bovine serum (Gibco, Grand Island, NY, USA) and antibiotics (100 U/mL penicillin and 100 μg/mL streptomycin) (Sigma-Aldrich, Shanghai, China) and kept at 37 °C with 5% CO_2_.

### 2.2. Effect of Quercetin and LPS on BIECs’ Viability

Applying a cell counting kit-8, the vitality of BIECs was evaluated (CCK-8; Vazyme, Nanjing, China). Quercetin (>95%) (Sigma-Aldrich, Shanghai, China) was mixed with DMSO and DMEM/F12 media to produce a 200 µg/mL stock compound, which was then refined by a 0.22 µm disinfected filter. The BIEC were seeded into 96-well plates (1 × 10^4^ cells per well) for 12 h and then treated with LPS or a series of concentrations of quercetin for 6 h. Subsequently, CCK8 was added into all of the experimental groups and incubated at 37 °C and 5% CO_2_ for 3 h. The OD values were acquired at 450 nm using a microplate reader.

### 2.3. Antioxidant Analysis

The concentration of BIECs in six-well growth plates was modulated to 1 × 10^6^ cells/mL. The cells proliferated to about 80–90% and were treated with LPS and quercetin. After 6 h of culture, these cells were collected. Total antioxidant capacity (TAC), malondialdehyde (MDA), glutonium peroxidase (GSH-Px), superoxide dismutase (SOD), and catalase (CAT) were evaluated in cell specimens by employing the Antioxidant Assay Kit (Jiancheng Bioengineering Institute, Nanjing, China) in accordance with the company’s guidelines. The TAC, MDA, GSH-Px, SOD, and CAT levels are given as U/mg protein relative to the density of cellular protein.

### 2.4. RNA Extraction, Reverse Transcription, and Real-Time PCR

RNA content was determined using an OD1000 machine (One drop 1000, Nanjing, China) after total RNA was derived from BIECs using TRIzol reagent (Takara, Code No. RR036A, China), per the package recommendations. Using an RT Kit, reverse transcription (RT) was performed (Takara, Beijing, China). The RT reaction compound included 1 μg of total RNA and 1 × PrimeScript RT Master Mix in an end quantity of 20 µL. The interactions were conducted for 15 min at 37 °C. Change transcriptase was heated to 85 °C for 5 s to inactivate it. qRT-PCR experiments were carried out with the SYBR^®^ Premix Ex TaqTM II Kit (Takara, Beijing, China). The qRT-PCR reaction mixture consisted of 1 × SYBR^®^ Premix Ex TaqTM II, 0.4 μM each forward and specific primers, and 100 ng cDNA formats in a final quantity of 20 µL, and responses were carried out as follows: first phase at 95 °C for 30 s, followed by 40 cycles at 95 °C for 5 s and 60 °C for 30 s. Standard dilution series were used to assess the production capabilities of all primers prior to performing qRT-PCR on samples. Table 1 contains the lists of the primers employed (made in GENEWIZ Bioscience Co. Ltd., Suzhou, China). The target genes were determined using the 2^−ΔΔCT^ approach after being normalised to that of GAPDH.

### 2.5. Immunofluorescence Analysis

At a concentration of 5 × 10^3^ cells/well, BIECs were developed on eight-well chamber slides. At about 80% agreement, they were treated with 100 µg/mL quercetin and 5 µg/mL LPS. The slides were then rinsed three times with PBS and kept with 4% paraformaldehyde at room temperature for 30 min, rinsed three times with PBS for 5 min each time, and then exposed to antigen recuperation with EDTA-Na_2_ (95 °C, 5 min). After exposure to 0.1% Triton X-100 (Sigma-Aldrich, St. Louis, MO) and three washes with PBS, the cells were closed using a blocking solution, including 3% horse serum. Following additional washes, phosphorylation-NF-B (p-p65) rabbit mAb (1:500; Cell Signaling Mechanism, Shanghai, China) was inserted and left overnight at 4 °C, followed by 30 min of exposure to goat anti-rabbit IgG conjugated with Cy3 (1: Beyotime Biotechnology Inc.). The cells were rinsed with PBS before being stained with DAPI for seven minutes. Immunofluorescence microscopy was performed using a confocal laser microscope (Olympus, Tokyo, Japan).

### 2.6. Western Blotting Analysis

Samples of cells for total protein separation were homogenised in RIPA buffer containing a protease suppressor (Thermo Scientific, Shanghai, China). Protein density was detected by employing BCA kits (Beyotime, Beijing, China). Using SDS-PAGE, equal quantities of protein lysates were separated and converted to nitrocellulose membranes (PALL, Shanghai, China). After blocking the membranes with 5% horse serum, they were placed overnight at 4 °C with the primary antibody and 5% horse serum in Tris-buffered saline with Tween (TBS-T: 10 mM Tris–HCl, pH 7.5, 150 mM NaCl, 0.05% Tween 20). The following main antibodies were collected from phosphorylation: NF-κB (p-p65) rabbit mAb (1:1000; Cell Signaling Technology, Shanghai, China), GAPDH (1:2000; Cell Signaling Technology, Shanghai, China), and p-IκBα rabbit mAb (1:1000; Affinity, Changzhou, China). Super Signal West Femto Maximum Sensitivity Substrate or Pierce ECL Plus Western Blotting Substrate was performed to recognize the target bands (Thermo Scientific).

### 2.7. Statistical Analysis

Data are illustrated as the means and the standard error of the mean (SEM). One-way ANOVA and Tukey’s multiple comparison tests were employed to identify major differences. The study’s data were analysed using the SPSS Statistics programme (IBM Corp., Armonk, NY, USA) version 19.0. A *p*-value < 0.05 denoted significance level, whereas *p* < 0.01 denoted highly significant results; 0.05 < *p* < 0.1 denoted trends.

## 3. Results

### 3.1. Impact of Quercetin on the Viability of BIECs

**Figure 1:**
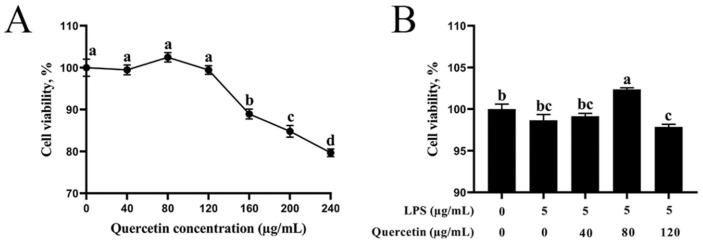
Impacts of quercetin on viable cells in LPS-induced BIECs. (**A**) Outcomes of quercetin on viable cells in BIECs. BIECs were treated with 40, 80, 120, 160, 200, or 240 µg/mL quercetin for 12 h. (**B**) Impacts of quercetin on cell vitality in LPS-induced BIECs. BIECs were given 5 µg/mL LPS and various densities of quercetin (40, 80, or 120 µg/mL) for 6 h. Records from the control group were employed to normalise the findings of each experimental group. The results are mean ± SEM (n = 6). Various lowercase letters in the line graph reveal significant differences (*p* < 0.05).

Initially, the impacts of various quercetin dosages on the stability of BIECs were evaluated. Figure 1A illustrates that quercetin reduced the viability of BIECs in a density manner. At values exceeding 120 μg/mL, there was a substantial decline. In this investigation, LPS activation did not affect the vitality of BIECs (Figure 1B). Furthermore, BIECs that received 5 μg/mL LPS were exposed to 40, 80, and 120 μg/mL of quercetin for 6 h. As seen in Figure 1B, therapy with 40 μg/mL quercetin and 5 μg/mL LPS had no effect on the vitality of BIECs versus the control group. With 80 μg/mL quercetin, the vitality of BIECs exposed to LPS was dramatically enhanced. Furthermore, 120 μg/mL of quercetin diminished the function of cells of LPS-treated BIECs (*p* < 0.05). As a result, 5 μg/mL LPS and 80 μg/mL quercetin were chosen as the therapeutic dose.

### 3.2. Effect of Quercetin on Oxidative Features of BIECs Influenced by LPS

**Figure 2:**
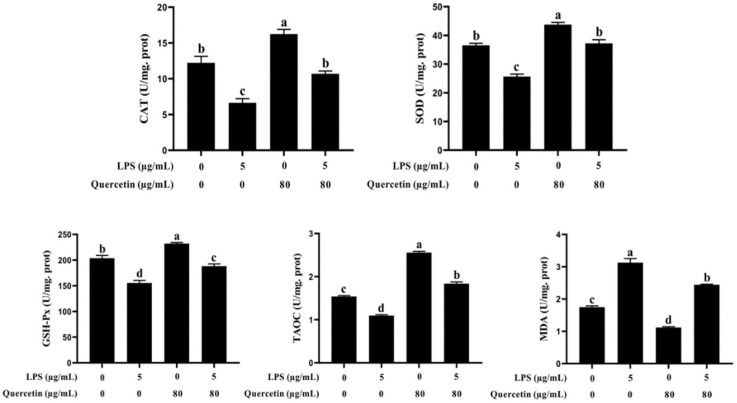
Impacts of quercetin on oxidative features in LPS-induced BIECs. BIECs were given 5 µg/mL LPS and 80 µg/mL quercetin. After 6 h of culture, the cells were gathered. The results are mean ± SEM (n = 6). Various lowercase letters in the line graph show significant changes (*p* < 0.05).

We further evaluated the capacity of quercetin to rescue BIECs from oxidative stress produced by LPS. The LPS-treated groups had lower levels of TAOC, SOD, CAT, and GSH-Px than the control group (*p* < 0.05, Figure 2). Nevertheless, the levels of TAOC, SOD, CAT, and GSH-Px were elevated in BIECs handled with quercetin only versus the control group (*p* < 0.05). Furthermore, quercetin elevated (*p* < 0.05) the levels of TAOC, SOD, CAT, and GSH-Px in LPS-activated BIECs compared with LPS alone. Moreover, the MDA level of BIECs handled with LPS was substantially higher than that of the control, but quercetin administration greatly lowered the MDA composition of LPS-treated BIECs (*p* < 0.05). These results revealed that quercetin was related to the antioxidant capacity of BIECs generated by LPS.

### 3.3. Effect of Quercetin on the Barrier Role of BIECs Caused by LPS

**Figure 3:**
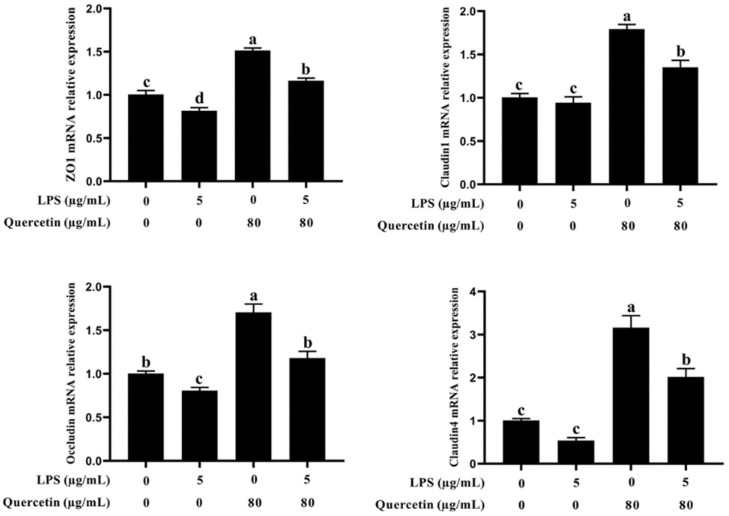
Impact of quercetin on barrier function in LPS-induced BIECs. BIECs were treated with 80 μg/mL quercetin in the environment of 5 μg/mL LPS for 6 h; The mRNA representation of zo-1, occluding, claudin 1, and claudin 4 in BIECs was examined by qRT-PCR. The results are mean ± SEM (n = 6). Various lowercase letters in the line graph reveal significant changes (*p* < 0.05).

To evaluate if quercetin has a beneficial influence on barrier action in LPS-treated BIECs, we examined the mRNA expression of zo-1, occluding, claudin 1, and claudin 4 using quantitative reverse transcription polymerase chain reaction (qRT-PCR). As illustrated in Figure 3, occludin mRNA expression was greatly diminished in LPS-treated groups versus the control group (*p* < 0.05, Figure 3). In addition, the mRNA expression of zo-1, occluding, claudin 1, and claudin 4 was substantially enhanced in BIECs handled with LPS plus 80 μg/mL quercetin compared with the LPS-treated group (*p* < 0.05, Figure 3). These findings indicate that quercetin protects against LPS-induced BIECs’ barrier breakdown.

### 3.4. Effect of Quercetin on Pro-Inflammatory Factors of BIECs Caused by LPS

**Figure 4:**
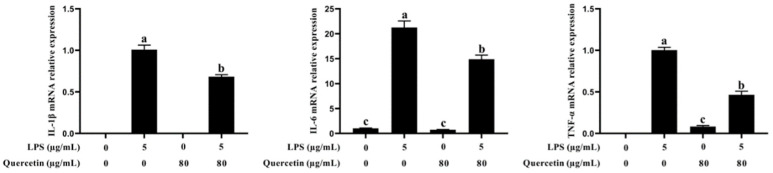
Impact of quercetin on the mRNA expression of pro-inflammatory cytokines (IL-1β, IL-6, and TNF-α) in LPS-induced BIECs. BIECs were treated with 80 μg/mL quercetin in the involvement of 5 μg/mL LPS for 6 h. The mRNA expressions of IL-1β, IL-6, and TNF-α in BIECs were examined by qRT-PCR. The results are mean ± SEM (n = 6). Various lowercase letters in the line graph demonstrate significant changes (*p* < 0.05).

qRT-PCR was used to assess the impact of quercetin on the generation of pro-inflammatory cytokines in LPS-activated BIECs by analysing genes associated with pro-inflammatory processes (Figure 4). mRNA expression of IL-1β, IL-6, and TNF-α was elevated in LPS-activated BIECs compared with the control group. TNF-α, IL-1β, and IL-6 mRNA expression was considerably lowered in LPS-treated BIECs with quercetin compared with the LPS-treated group (*p* < 0.05, Figure 4). Such results suggest that quercetin can suppress the production of pro-inflammatory cytokines in BIECs stimulated with LPS.

### 3.5. Effect of Quercetin on the Immune Reaction of BIECs Caused by LPS

**Figure 5:**
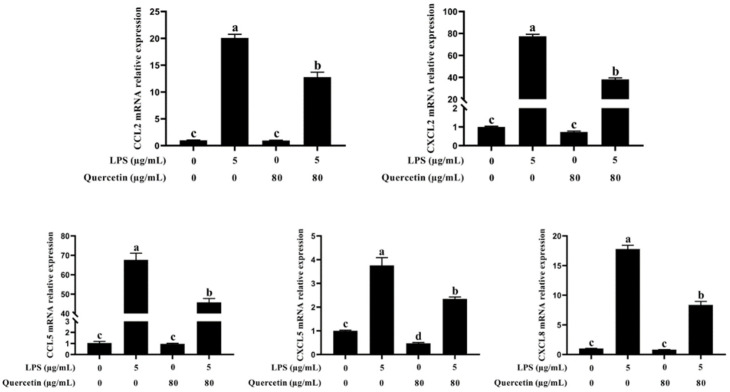
Quercetin’s effect on the mRNA expression of chemokines in LPS-stimulated BIECs. BIECs were treated with 80 μg/mL quercetin in an environment of 5 μg/mL LPS for 6 h. The mRNA expressions of CCL2, CCL5, CXCL2, CXCL5, and CXCL8 in BIECs were measured by qRT-PCR. The results are mean ± SEM (n = 6). Various lowercase letters in the line graph reveal significant changes (*p* < 0.05).

CCL2, CCL5, CXCL2, CXCL5, and CXCL8 mRNA expression was considerably increased in LPS-treated BIECs compared with untreated cells (*p* < 0.05, Figure 5). In contrast, the development of immune response-related genes CCL5, CXCL2, CXCL5, and CXCL8 was lower in BIECs treated with 80 μg/mL quercetin and 5 μg/mL LPS (*p* < 0.05, Figure 5). These findings demonstrated that quercetin modulated the immune function elicited by LPS in BIECs.

### 3.6. Effect of Quercetin on the TLR4 Signalling Pathway of BIECs Caused by LPS

**Figure 6:**
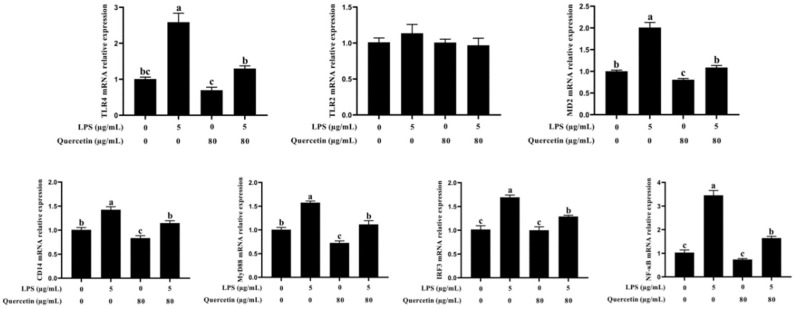
Influence of quercetin on the TLR4 signalling system in LPS-induced BIECs. BIECs were treated with 80 μg/mL quercetin in an environment of 5 μg/mL LPS for 6 h. The mRNA expressions of TLR4, TLR2, MD2, CD14, MyD88, IRF3 and NF-κB in BIECs were tested by qRT-PCR. The results are mean ± SEM (n = 6). Various lowercase letters in the line graph demonstrate significant changes (*p* < 0.05).

qRT-PCR was used to evaluate the expression of TLR4, CD14, MD2, MyD88, NF-κB, and IRF3 genes implicated in the TLR4 signalling system. As seen in Figure 6, the mRNA levels of TLR4, CD14, MD2, MyD88, NF-κB, and IRF3 were substantially elevated in LPS-treated BIECs, but greatly lowered in LPS-induced BIECs handled with 80 μg/mL quercetin (*p* < 0.05, Figure 6). Figure 6 demonstrates that quercetin and LPS have no significant influence on the expression of TLR2 (*p* > 0.05, Figure 6). Our results indicated that quercetin inhibits the TLR4 signalling mechanism to reduce pro-inflammatory actions caused by LPS in BIECs.

### 3.7. Effect of Quercetin on the NF-κB Signalling Mechanism of BIECs Caused by LPS

**Figure 7:**
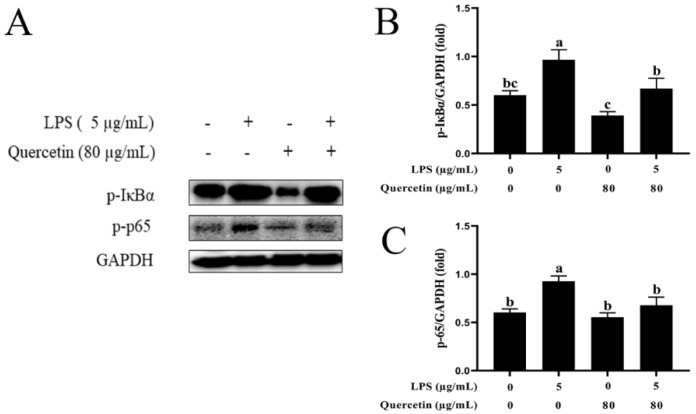
Impacts of quercetin on the NF-κB signalling system. BIECs were treated with 80 μg/mL quercetin in an environment of 5 μg/mL LPS for 6 h. (**A**) Western blot evaluation of p-IκBα and p-p65. (**B**) Relative protein expression levels of p-IκBα to GAPDH. (**C**) Relative protein expression levels of p-p65 to GAPDH. The results are mean ± SEM (n = 3). Various lowercase letters in line graphs reveal significant changes (*p* < 0.05).

**Figure 8:**
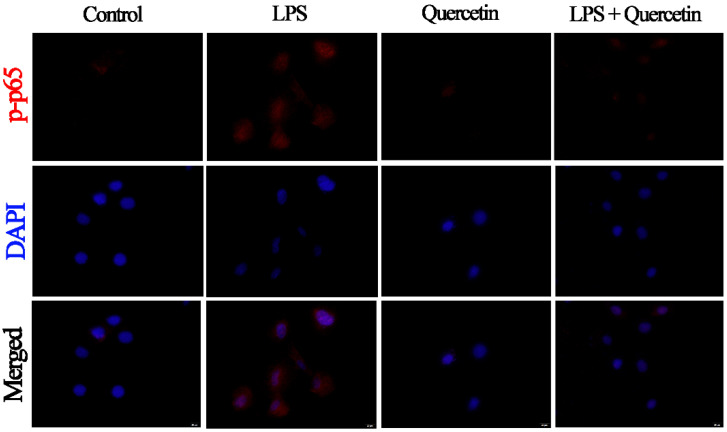
Impact of quercetin on the NF-κB (p65) signalling mechanism in LPS-induced BIECs. BIECs were treated with 80 μg/mL quercetin in an environment of 5 μg/mL LPS for 6 h. The immunofluorescence for phosphorylation-NF-κB (p-p65) (red) was conducted and the nuclear dye 4′, 6-diamidino-2-phenylindole (DAPI; blue) was included. Scale bar = 20 µm.

LPS administration raised p-IB/GAPDH (*p* < 0.05) compared with the control group. In addition, stimulation with quercetin lowered p-IκBα/GAPDH compared with the LPS-treated group (*p* < 0.05, Figure 7A,B). Similarly, LPS administration upregulated p-p65/GAPDH (*p* < 0.05), but quercetin administration suppressed the LPS-induced elevation of p-p65/GAPDH (*p* < 0.05, Figure 7A,C). Immunofluorescence analysis revealed that LPS triggers the NF-κB system via TLR4 in BIECs, resulting in elevated levels of p-p65. In addition, we discovered that LPS activation substantially boosted the nuclear entrance of p-p65 in BIECs, but quercetin administration suppressed this nuclear entry (Figure 8).

## 4. Discussion

It is believed that BIECs serve an essential function in preventing the entrance of harmful microorganisms [23]. Research has indicated that pathogenic bacteria stimulate the gut region with high levels of LPS and that persistent LPS can worsen the inflammatory processes of the intestines [24]. LPS, a significant element of the exterior surface of gram-negative bacteria, stimulate a robust immune reaction in affected tissues [25].

High amounts of LPS may result in oxidative stress due to a redox disequilibrium in the cell [24]. Extreme oxidative tension triggers immune cells to initiate the sequence of the inflammatory process [25]. Existing research implies that inflammatory processes and oxidative stress interact. Inflammatory cells possess reactive oxygen species (ROS) to contribute to oxidative tension, but ROS can promote the production of inflammatory cytokines [26]. Furthermore, intestinal epithelial cells were implicated in the creation of the intestinal barrier, which protects the efficiency of digestion in animals [27]. Earlier studies have shown that intestinal inflammation may modify the accessibility of the intestinal barrier and that intestinal barrier failure exacerbates and maintains intestinal inflammation [28]. This barrier malfunction may be the consequence of changes in junctional protein [29]. As a result, it is important to find potential nontoxic replacements for LPS-induced pro-inflammatory processes and oxidative overload in order to improve barrier action and trigger innate immune responses, which will help LPS infectious disease heal faster.

Currently, plant parts with anti-inflammatory and anti-inflammatory chemicals are a secure and efficient method for treating intestinal inflammation in cattle. It has been established that quercetin possesses anti-inflammatory and antioxidant features [14,30]. In vitro and in vivo studies have illustrated that quercetin shields epithelial cells from oxidative destruction [31] and lowers paraquat caused by oxidative injury via controlling the activity of antioxidant genes in cells [32]. Quercetin ameliorated LPS caused by inflammation in HGFs by stimulating PPAR-γ and lowering the stimulation of NF-κB, according to Xiong et al. [15]. Various research has shown the medicinal qualities of quercetin in the treatment of inflammatory diseases [33,34,35,36]. Unfortunately, the regulating method of quercetin on anti-inflammatory, antioxidant, and barrier function in LPS-induced BIECs remains unclear at this time. Throughout this experiment, we examined the protective properties of quercetin on the anti-inflammatory, antioxidant, and barrier function in experimental LPS-induced BIECs. LPS interacts with TLR4, causing the activation of the NF-κB signal system and the formation of reactive oxygen species to control the development of antioxidant enzyme activities [37]. Within that work, we evaluated the levels of SOD, GSH-Px, TAOC, MDA, and CAT in BIECs generated by LPS. With quercetin administration, the levels of SOD, GSH-Px, TAOC, and CAT were substantially increased in LPS-induced BIECs compared with LPS only. Furthermore, the administration of quercetin substantially lowered MDA levels. As predicted, LPS content reduces these antioxidant enzyme markers, which is similar to the present findings [38]. Thus, our findings confirmed that quercetin raised the levels of SOD, CAT, GSH-Px, and TAOC and lowered the amount of MDA in LPS-induced BIECs, thus reducing the pro-inflammatory characteristics of BIECs.

Next, we investigated the role of quercetin in the LPS-induced impairment of barrier function in BIECs. The inflammatory cytokines released by cells in reaction to LPS compromise the function of the epithelial barrier, hence aggravating epithelial injury and inflammation [39]. The preservation of intercellular tight junction production is a key predictor of epithelial barrier integrity and paracellular permeability, according to previous research [40]. qRT-PCR was employed to assess the mRNA expression of epithelial tight-junction protein markers (zo-1, occludin, claudin 1, and claudin 4). Occludin mRNA expression was considerably lowered in the LPS-treated group compared with the control group, indicating impaired tight junction stability. These findings revealed that quercetin had protective properties toward LPS-induced BIEC barrier breakdown.

To investigate the anti-inflammatory abilities of quercetin, we examined its effect on BIECs exposed to LPS. TLR4 is a sensor for pattern recognition [41]. TLR4 can recognize LPS and then activate the NF-κB signaling pathway, which stimulates p-p65 protein accumulation in the nucleus and the subsequent induction of inflammatory responses, promoting the secretion of inflammatory cytokines, such as TNF-α, IL-6, and IL-1β [42,43]. Another researcher has also discovered that TLR4 triggers MyD88 and, afterwards, causes the stimulation of the NF-κB and MAPK signalling mechanisms, hence increasing the production of pro-inflammatory cytokines [44]. Through the creation of chemokines and attached molecules [45], BIECs can activate the immune system in response to invading pathogens. Cytokines and chemokines make crucial contributions in directing LPS-mediated immune function [46]. The primary function of chemokines is to attract many immune cells to the site of tissue localization; these cells then produce numerous active substances that contribute to tissue’s immunological damage and inflammatory response. When comparing the LPS group to the control group, the chemokines CXCL2, CXCL5, CCL5, and CXCL8 were substantially increased in the LPS group. This demonstrates that LPS can stimulate the production of chemokines and the enhanced expression of chemokines can facilitate the migration of chemotactic immune cells to the mammary gland immune layer to modulate the LPS. In addition, the expression of chemokines was considerably reduced in the quercetin and LPS + quercetin groups compared with the LPS group. Furthermore, LPS activation substantially raised the mRNA levels of pro-inflammatory cytokines (TNF-α, IL-6, and IL-1β) in BIECs, but quercetin administration inverted this trend, demonstrating that quercetin might limit LPS-induced inflammation reactions in BIECs. Likewise, the associated genes of the TLR4 signalling pathway confirmed that the mRNA expressions of TLR4, CD14, MD2, MyD88, and IRF3 were substantially elevated in LPS-treated BIECs, but greatly lowered in LPS-induced BIECs treated with quercetin. To corroborate this conclusion further, Western blotting was performed on the NF-κB signalling system and compared to immunofluorescence. The LPS treatment enhanced the proportions of p-IκBα/GAPDH and p-p65/GAPDH. Compared with the LPS-treated group, quercetin administration lowered the proportions of p-IκBα/GAPDH and p-p65/GAPDH. Moreover, immunofluorescence data revealed that LPS activation greatly boosted the nuclear entrance of p-p65 in BIECs, but quercetin administration prevented this effect in LPS-induced BIECs. These results revealed that quercetin inhibits the TLR4-NF-κB signalling mechanism to suppress the immune reactions of BIECs caused by LPS. In the meantime, these findings imply that quercetin could be useful for treating intestinal inflammation.

## 5. Conclusions

The present study has proved that quercetin can effectively alleviate oxidative stress and damage to the barrier function of BIEC induced by LPS in vitro. It can be seen that quercetin may be a promising bioactive extract for protecting the natural biological performance of BIECs and alleviating the negative effects of LPS. Therefore, in production, we can improve the health status and performance of dairy cows by adding quercetin to the diet to reduce the effects of intestinal inflammation.

## Figures and Tables

**Table 1:** Primers for real-time quantitative PCR.

Gene	Primer Sequence ^1^, 5′ to 3′	Source	Size (bp)
GAPDH	F: GGGTCATCATCTCTGCACCT R: GGTCATAAGTCCCTCCACGA	NM_001034034.2	176
IL-1β	F: CAGTGCCTACGCACATGTCT R: AGAGGAGGTGGAGAGCCTTC	NM_174093.1	209
IL-6	F: CACCCCAGGCAGACTACTTC R: TCCTTGCTGCTTTCACACTC	NM_173923.2	129
TNF-α	F: GCCCTCTGGTTCAGACACTC R: AGATGAGGTAAAGCCCGTCA	NM_173966.3	192
CXCL5	F: TGAGACTGCTATCCAGCCG R: AGATCACTGACCGTTTTGGG	NM_174300.2	193
CCL5	F: CTGCCTTCGCTGTCCTCCTGATG R: TTCTCTGGGTTGGCGCACACCTG	NM_175827	217
CCL2	F: GCTCGCTCAGCCAGATGCAA R: GGACACTTGCTGCTGGTGACTC R: GCACAACTTGTTTCACCCACT	NM_174006	171
CXCL2	F: CCCGTGGTCAACGAACTGCGCTGC R: CTAGTTTAGCATCTTATCGATGATT	NM_174299.3	204
CXCL8	F: TGGGCCACACTGTGAAAAT R: TCATGGATCTTGCTTCTCAGC	NM_173925.2	136
ZO-1	F: TCTGCAGCAATAAAGCAGCATTTC R: TTAGGGCACAGCATCGTATCACA	XM_010817146.1	187
Occludin	F: GAACGAGAAGCGACTGTATC R: CACTGCTGCTGTAATGAGG	NM_001082433.2	122
Claudin 1	F: CGTGCCTTGATGGTGAT R: CTGTGCCTCGTCGTCTT	NM_001001854.2	102
Claudin 4	F: CTTCATCGGCAGCAACATC R: ACAACAGCACGCCAAACA	NM_001014391.2	191
TLR2	F: CAGGCTTCTTCTCTGTCTTGT R: CTGTTGCCGACATAGGTGATA	NM_174197.2	140
TLR4	F: GACCCTTGCGTACAGGTTGT R: GGTCCAGCATCTTGGTTGAT	NM_174198.6	103
CD14	F: CAGTATGCTGACACAATCAA R: AGTTCCTTGAGACGAGAGTA	NM_174008.1	122
MD2	F: GGAGAATCGTTGGGTCTGC R: GCTCAGAACGTATTGAAACAGGA	NM_001046517.1	92
MyD88	F: TCATTGAGAAGAGGTGCCGT R: TGGCTTGTACTTGATGGGGAT	NM_001014382.2	146
IRF3	F: TTGTGAACTCAGGAGTCAGG R: TGGGCTCAAGTCCATGTCAC	NM_001029845.3	125

^1^ F, forward; R, reverse.

## Data Availability

The datasets given in this research are accessible from the corresponding author upon request.
